# Contrasting spatial and temporal activity patterns of little brown bats (*Myotis lucifugus*) at maternity roosts and swarming sites

**DOI:** 10.1093/jmammal/gyag001

**Published:** 2026-02-05

**Authors:** Jade Legros, Liam P McGuire, Anouk Simard, Kyle H Elliott

**Affiliations:** Department of Natural Resource Sciences, McGill University, 21111 Lakeshore Road, Ste. Anne de Bellevue, Montreal, QC, H9X 3V9, Canada; Department of Biology, University of Waterloo, 200 University Avenue West Waterloo, Ontario, N2L 3G1, Canada; Ministère de l’Environnement, de la Lutte contre les changements climatiques, de la Faune et des Parcs, 880 Ch Ste-Foy, Québec, QC, G1S 4X4, Canada; Department of Natural Resource Sciences, McGill University, 21111 Lakeshore Road, Ste. Anne de Bellevue, Montreal, QC, H9X 3V9, Canada

**Keywords:** automated radiotelemetry, central place, hibernaculum, Little Brown Bat, maternity roost, swarming

## Abstract

Life history of many animals is often concentrated around central or focal places such as roosts or breeding sites that impose spatial constraints and shape habitat use. Central place behaviors vary depending on life stages, environmental conditions, and the annual cycle of a species—highlighting the need to consider these variations in management plans. The objective of our study was to contrast central place behaviors of little brown bats (*Myotis lucifugus*) during summer around a maternity roost, while lactating females raised their pups, and during pre-hibernation swarming (mating) at a cave for both males and females. Given the differences in energetic constraints between seasons and sexes, we hypothesized that return rates, activity, distribution, and habitat use would differ between the maternity roost and the hibernaculum and that male and female behavior would differ. We used an automated telemetry network consisting of 10 receiver towers at each site to track bat movements in the surrounding area. This system recorded over 370,000 detections from 23 lactating females at the maternity roost and over 90,000 detections from 23 males and 15 females at the hibernaculum. The maternity roost acted as a typical central place with females returning to the roost on 81 ± 29% of nights and with activity concentrated in a ∼5 km radius of the roost, primarily along a riparian corridor. At the swarming site, males and females returned on only 22 ± 27% of nights, although when they returned males stayed longer than females. Bats were detected up to 13 km from the swarming site and males were overall detected more frequently than females, suggesting that the spatial extent of use differs between sexes. Our study is one of the first to automatically track bats around both a maternity roost and a hibernaculum, and we offer practical suggestions to improve study designs—particularly to address seasonal shifts and sex-specific behavioral variation. We also highlight the importance of integrating seasonal perspectives to better support ­management plans for bats.

## Patrones de actividad espacial y temporal contrastantes de murciélagos en refugios de maternidad y sitios de enjambre

El ciclo de vida de muchos animales suele concentrarse en lugares centrales o focales, como dormideros o zonas de reproducción, que imponen restricciones espaciales y condicionan el uso del hábitat. Los comportamientos en lugares centrales varían según las etapas de la vida, las condiciones ambientales y el ciclo anual de la especie, lo que resalta la necesidad de considerar estas variaciones en los planes de gestión. El objetivo de nuestro estudio fue comparar los comportamientos en lugares centrales del murciélago pardo pequeño (*Myotis lucifugus*) durante el verano alrededor de un dormidero de maternidad mientras las hembras lactantes crían, y durante el enjambre (apareamiento) prehibernativo en una cueva, para ambos machos y hembras. Dadas las diferencias en las restricciones energéticas entre estaciones y sexos, formulamos la hipótesis de que las tasas de retorno, la actividad, la distribución y el uso del hábitat diferirían entre el dormidero de maternidad y el hibernáculo, y que el comportamiento de machos y hembras sería diferente. Utilizamos una red de telemetría automatizada compuesta de diez torres receptoras en cada sitio para rastrear los movimientos de los murciélagos en el área circundante. Este sistema registró más de 370,000 detecciones de 23 hembras lactantes en el dormidero de maternidad y más de 90,000 detecciones de 23 machos y 15 hembras en el hibernáculo. El dormidero de maternidad actuó como un lugar central típico con hembras regresando al dormidero 81 ± 29% de las noches y con actividad concentrada en un radio de ∼ 5 km del dormidero, principalmente a lo largo de un corredor ribereño. En el sitio de enjambre, los machos y las hembras regresaron solo 22 ± 27% de las noches, aunque cuando regresaban, los machos se quedaban más tiempo que las hembras. Detectamos murciélagos hasta a 13 km del sitio de enjambre. En general, los machos se detectaban con mayor frecuencia que las hembras, lo que sugiere que la extensión espacial de uso difiere entre sexos. Nuestro estudio es uno de los primeros en rastrear automáticamente a los murciélagos tanto en sus zonas de maternidad como en sus zonas de hibernación, y ofrecemos sugerencias prácticas para mejorar los diseños de los estudios, en particular para abordar los cambios estacionales y la variación conductual específica según el sexo. También destacamos la importancia de integrar las perspectivas estacionales para respaldar mejor los planes de gestión de murciélagos.

**palabras clave:** dormilón de maternidad, enjambre, hibernáculo, lugar central, murciélago pardo pequeño, radiotelemetría automatizada

As demands, costs, and constraints vary through the annual cycle or with environmental variation, animals move among different places that provide food, mates, safety from predators, or favorable microclimates. Many animals depend on central places such as a residence (roost, den, nest, etc.) or other focal sites (e.g., mating sites, water sources), which create spatial constraints that dictate the relative value and availability of habitat in the areas surrounding the central or focal place (Rosenberg and McKelvey 1999; Rainho and Palmeirim 2011). The benefit of visiting a habitat patch must outweigh the cost of commuting between the patch and the central place ­(Orians and Pearson 1979; Olsson et al. 2008; Pyke 2010). In the scenario where an animal often returns to its central place (e.g., parental care of non-mobile offspring), the relative value of closer habitat patches increases because nearby habitats have lower commuting costs (Olsson and Bolin 2014). For example, the foraging range of Purple Martin (*Progne subis*) is 100 times larger at winter roosts that they visit only once per day than during the breeding season when adults must provision chicks at the nest many times a day (Lalla et al. 2022).

The variable constraints of central place foraging means that the degree of habitat selection is likely to vary throughout the annual cycle, as individuals constrained to forage near the central place may reduce commuting costs by lowering habitat selectivity (e.g., Lamb et al. 2020; O’Neill et al. 2020; Stanley et al. 2021). Although during migration, songbirds tend to be more generalist in terms of habitat requirements, relatively to breeding seasons when they select specific habitats that fulfill needs related to parental care (e.g., Stanley et al. 2021). Throughout the annual cycle, environmental constraints linked to food availability or physical limitations also modulate behavior. For example, White-tailed Deer (*Odocoileus virginianus*)—in the northern part of their range—select forested winter habitats that limit snow cover and reduce movement cost, while in the summer they prefer open habitats with abundant forage opportunities (Beier and McCullough 1990).

Several studies have considered central place behaviors of bats during the summer reproductive period (e.g., Daniel et al. 2008; Rainho and Palmeirim 2011; Fahr et al. 2015). Females of many species gather at maternity roosts for the period of pregnancy and lactation (e.g., Henry et al. 2002). In contrast, the behavior of bats around hibernacula during the swarming period has received less attention, and most existing studies have focused on males (e.g., Lowe 2012; Perry et al. 2016). In winter, hibernacula (e.g., caves, mines) serve as seasonal residences for many species of hibernating bats (e.g., McGuire et al. 2009; Gallant and Broders 2015). Prior to hibernation, bats visit these sites in a behavior known as swarming but do not commonly roost in the cave/mine during the day in early swarming season (Fraser and McGuire 2023), although males are known to roost in the vicinity (Lowe 2012; Perry et al. 2016). Thus, during the pre-hibernation season swarming sites are used as central places mostly for mating, but behavior around these sites remains largely unknown, especially for females that are underrepresented in captures at these sites (Lowe 2012; Burns and Broders 2015; Perry et al. 2016). Sex-specific energetic constraints, habitat requirements, and central place demands during the maternity and swarming periods likely shape distinct spatial and temporal activity patterns. Understanding variation in habitat use is an important question for understanding animal ecology, with important applied consequences for management practices of threatened and endangered species (e.g., Lamb et al. 2020; Lalla et al. 2022).

We contrasted Little Brown Bat (*Myotis lucifugus*) central place behavior at 2 periods of the annual cycle—at a maternity roost and at a pre-hibernation swarming site. Because females need to nurse pups on a nightly basis, we hypothesized that females would have a high revisitation rate (return every night) at the maternity roost, and that activity would be concentrated in habitats close to the roost. At the swarming site during the late summer mating season, we expected that bats trade off time and effort invested in mating and foraging activities prior to hibernation (time spent at the swarming site for mating, time spent away from the swarming site for pre-hibernation fattening). Consequently, we predicted that bats would have a relatively low revisitation rate compared to the maternity colony if bats only commuted to the swarming site to participate in mating activities on some nights; alternatively, bats could commute to the hibernaculum every night to mate and forage nearby. However, energetic trade-offs should be especially pronounced for females with depleted fat stores following lactation (Knuz et al. 1998), and we therefore hypothesized sex-specific central place behavior at the swarming site for this promiscuous species (Thomas et al. 1979). Frequent returns to the swarming site to increase mating opportunities might benefit males more than females (Burns and Broders 2015), as females may prioritize per-hibernation fattening to recover from the energetic demands of summer reproduction (Jonasson and Willis 2012). At both sites, we predicted that activity would be concentrated in high-quality habitat (see Table 1 for detailed predictions).

**Table 1.** Habitat features covered by receiver towers. Descriptions, mechanisms, and their predicted effects on Little Brown Bat activity around a maternity roost (June to July 2021) and a swarming site (September 2021) in the Outaouais region, Québec, Canada.

Habitat features	Predicted effect	Description	Mechanism	References
**Distance to central place**	-	Minimum Euclidian distance to the maternity roost/Swarming site	Space use is constrained by the distance to the site	(Orians and Pearson 1979; Rosenberg and McKelvey 1999)
**Distance to river (maternity only)**	-	Minimum Euclidian distance to the Désert River	Bats use linear features such as rivers to commute and forage	(Furmankiewicz and Kucharska 2009; Rainho and Palmeirim 2011)
**% Young forest**	-	% forest stand <80 yr old	Bats avoid young forest because it is too cluttered	(Thomas et al. 2021)
**% Old forest**	+	% forest stand >80 yr of	Bats prefer to commute and forage in old forest because it is less cluttered and potentially supports more insects	(Crampton and Barclay 1998; Law et al. 2016)
**% Water**	+	% cover of water	Little brown bats forage over water	(Bergeson et al. 2013; Kniowski and Gehrt 2014)
**% Wetlands**	+	% cover of wetland	Wetlands provide high abundance and diversity of insects where bats can forage	(Coleman et al. 2014; Lookingbill et al. 2010)
**% Urban**	-	% cover of human modified surface (including roads, urban and industrial cover)	Little brown bats avoid foraging in human-modified habitat	(Fabianek et al. 2011; Krauel and LeBuhn 2016)
**% Harvested**	-	% harvested forest in the last 15 yr	Bat activity decreased in harvested forest patches	(Grindal and Brigham 1999; Patriquin and Barclay 2003)
**% Agriculture**	-	% cover of all types of agriculture	Little brown bats avoid open spaces like agriculture	(Kniowski and Gehrt 2014; Thomas et al. 2021)
**Water edges**	+	Density of water edges	Bats use water edges for commuting and foraging	(Holloway and Barclay 2000; Ford et al. 2005)
**Forest edges**	+	Density of forest edges	Bats use forest edges for commuting and foraging	(Grindal and Brigham 1999; Patriquin and Barclay 2003)

## Methods

### Study sites.

**Fig. 1.**
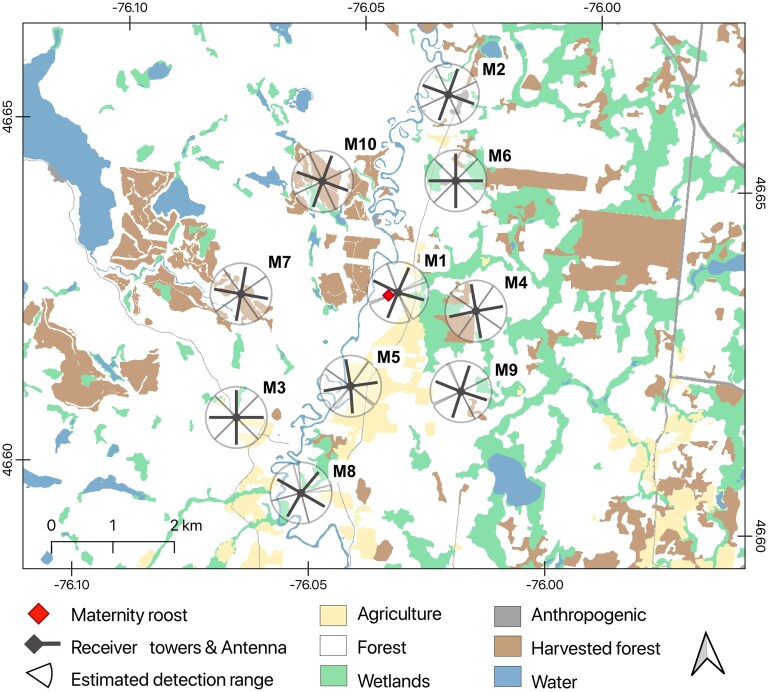
Study area and receiver tower locations around the maternity roost (Montcerf-Lytton) in the Outaouais region, Québec, Canada.

**Fig. 2.**
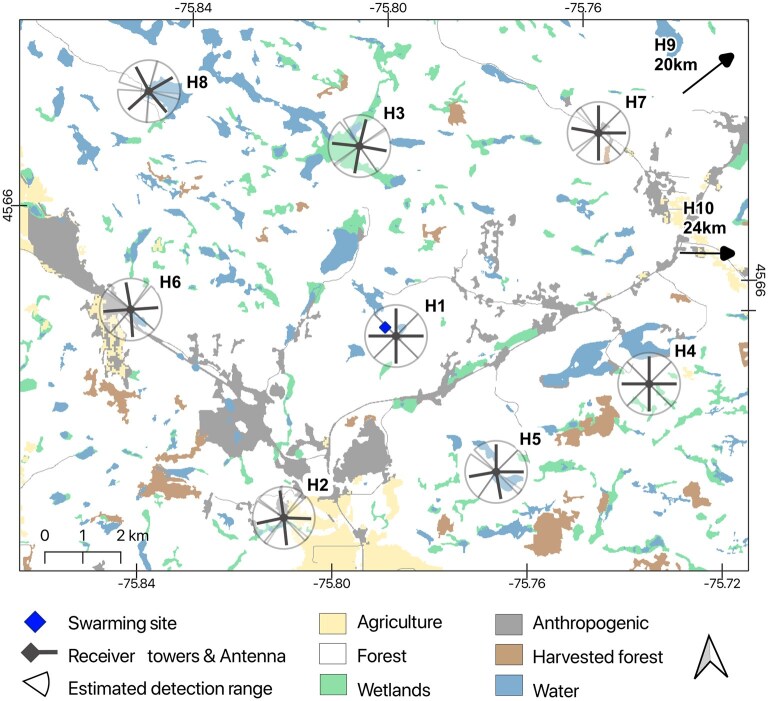
Study area and receiver tower locations around the swarming site (Val-des-Monts) in the Outaouais region, Québec, Canada.

Our study was conducted at a maternity roost in a house and at a cave hibernaculum, about 110 km apart in the Outaouais region of Québec, Canada (Figs 1 and 2). White-nose syndrome was first recorded in this region in 2010, but it was among the first regions in Québec where increasing population size was observed following the initial 70% to 90% decline. The maternity colony occupied the roof and walls of a house since 2011 according to the owner of the house (Montcerf-Lytton, Québec, approximate location [46.60°N, 76.00°W]; Fig. 1). In 2021, we conducted emergence surveys on 15 and 30 June, when females are thought to be lactating and counted 141 and 147 bats, respectively, mainly little brown bats based on capture data and acoustic monitoring during bat emergence. In June and July 2021, the highest temperature recorded in the area was 24 °C and the lowest temperature 11 °C, and precipitation summed to 63 mm per month (Environment and Climate Change Canada 2025).

The hibernaculum site, Laflèche Cave (Val-des-Monts, Québec [45.65°N, 75.79°W]), is a year-round commercially-operated tourist cave. In March 2021, 468 bats were counted hibernating in the cave, mostly little brown bats or northern long-eared bats (*M. septentrionalis*). Laflèche Cave is among the few remaining hibernacula in the province with >100 bats since widespread population declines from white-nose syndrome. Bats fly into and out of the cave from 1 known entry, next to a small lake (Fig. 2). In September 2021, the highest temperature recorded in the area was 21 °C and the lowest temperature 10 °C, and precipitation summed to 148 mm (Environment and Climate Change Canada 2025).

### Automated and manual radiotelemetry.

We captured bats using harp traps and mist nets on 20 June 2021 at the maternity roost during the lactation period, and on 31 August 2021 at the hibernaculum during the swarming period (Table 2). We noted body mass (digital scale; ±0.01 g), sex, reproductive status (pregnant, lactating, post-lactating, non-reproductive), age (adult or subadult; determined by the degree of ossification of the metacarpal–phalanges joint; Davis and Hitchcock 1965), and forearm length as a measure of body size (digital calipers ±0.01 mm). We banded and attached digitally-coded radio transmitters (hereafter “tags”; Nanotag NTQB2-1, 0.26 g < 4% of body mass; Lotek Wireless, Newmarket, Ontario, Canada) to adult bats only with GLUture topical adhesive (Zoetis Inc, Kalamazoo, Michigan) after trimming the fur in the intrascapular dorsal region. All tags were encoded on the same frequency but had a unique digital signature allowing simultaneous monitoring. Radio tags had a pulse rate of 5.3 s for a total expected battery life of approximately 1 mo. All research activities were conducted under a protocol from the ministère de l’Environnement, de la Lutte contre les changements climatiques, de la Faune et des Parcs (MELCCFP; #21-16) and approved by both the McGill University Animal Care Committee and the MELCCFP Animal Care Committee. In the context of COVID-19, Canadian Wildlife Health Cooperative guidelines were followed (Canadian Wildlife Health Cooperative 2021).

**Table 2.** Timeline of field activities at the maternity roost and swarming site in 2021, Outaouais region, Québec, Canada.

Site	Period associated^a^	Tower installation	Bat capture and tagging	Latest bat detections	Tower removal
**Maternity roost**	Pup/lactation season June 1 to August 15	June 15 to 17	June 20	July 8	July 21
**Swarming site**	Swarming season August 16 to October 31	August 24 to 26	August 31	September 27	September 27

a Informed by Quebec acoustic survey protocol periods (ministère de l’Environnement, de la Lutte contre les changements climatiques, de la Faune et des Parcs 2023) and by bat activity dates from closest USFS forest service (Green Mountain and Finger Lakes NF forest service; US Forest Service and US Fish and Wildlife Service 2024).

We tracked bats using automated telemetry with fixed receiver stations (hereafter “towers”), complemented with manual mobile telemetry. Towers consisted of a datalogging telemetry receiver (SRX800 or SRX1200; Lotek Wireless) connected to 4 5-element Yagi antennas (Lotek Wireless) mounted at a height of 4 m, with a switch box that cycled through each antenna every 8 s. We installed an array of towers (10 at the maternity roost or 8 at the hibernaculum) that represented a radius of 4.5 km around the maternity roost and 6 km around the hibernaculum (Figs 1 and 2). During swarming, we installed 2 additional towers at the entry of known hibernacula in the vicinity of our study cave: Emerald Mine (24 km away) and High Rock Mine (20 km away; Fig. 2). To monitor return activity, we installed 1 tower as close as possible to the entry of the maternity roost (<10 m; identified as M1 in Fig. 1) and the hibernaculum (<100 m; identified as H1 in Fig. 2). The location of surrounding towers throughout the landscape mainly followed a uniform distribution to maximize landscape coverage and minimize zones without coverage. We estimated that each tower had a detection range of about 500 m (Supplementary Data SD1). We then selected specific sites to diversify habitat types, constrained by limited access on private land (Table 2).

The goal of automated telemetry is not to identify precise locations of animals as in manual telemetry methods based on biangulation or triangulation. Rather, automated telemetry tracks approximate locations across a broad study area, as towers may detect bats even from a considerable distance (>3 km; Supplementary Data SD1). Relative signal strength and detections on multiple antennas provide further context for individual locations. For example, if a bat is close enough to a tower, all 4 antennas should detect it regardless of its position, increasing the rate of detection when bats are close. Although antennas can detect signals at angles >90°, the probability of detection decreases substantially between 90° and 180° offset angles (Crewe et al. 2019).

To supplement the automated telemetry, we opportunistically tracked bats using mobile receivers and handheld 3-element Yagi antennas, and searched for day roosts during the swarming period only as we suspected bats would not necessarily roost inside the cave during this period. Following roads inside and outside the detection range of towers, we randomly scanned the landscape for at least 5 min at fixed locations where the habitat seemed favorable. We made 44 manual scans within a 5.7 km radius of the maternity roost over a period of 8 nights (between 21 June and 6 July). For the hibernaculum, we increased to 278 manual telemetry scans within a 14.5 km radius of the hibernaculum during 12 nights and days (between 31 August and 20 September). If a bat was detected during the day, we triangulated its position, when possible, but did not track bats to roosts because of limited access to private properties. We used manual telemetry data to evaluate the approximate area used around both sites and to complement automated telemetry detections.

### Automated radiotelemetry data cleaning.

We filtered all detections collected from towers following best practices (Crewe et al. 2018), considering only observations with 3 or more consecutive detections at 1 tower (run length ≥3; Crewe et al. 2018). We considered consecutive detections as separated by <40 s, accounting for the time required to cycle through all antennas of a tower and the tag pulse rate. This method reduces the likelihood of false positive detections (Crewe et al. 2018). Although some true detections may have been excluded in the process, it helps ensure that the data reflect actual space use around the tower rather than brief transit events.

We estimated the minimum number of nights tags remained attached to bats (hereafter “active nights”) by assuming a tag fell off when we detected regular detection with consistent power at 1 tower, or when there were no further detections. Data cleaning and all subsequent analyses were performed in R (version 4.2.2; R Core team 2022).

### Return rate and visitation times.

We calculated the nightly return rate based on detections from towers closest to each site (M1 and H1; Figs 1 and 2). We defined a return to the maternity or the swarming site by a batch of consecutive detections of a bat at any antenna of the central tower (M1 and H1) during at least a 2-min window. A 2-min window was selected arbitrarily, the time itself being less important than the number of visits for the purpose of spatial dependency study. We calculated the return rate as the number of nights a bat returned to the site (whatever the purpose of the return such as resting, nursing, mating, foraging, etc.) based on the number of active nights of each bat, excluding bats with only 1 active night. We used logistic regression to compare the return rate between sites and sexes.

When bats were detected at the central place, we evaluated the visitation time (how much time a bat was detected by the central tower during the night) at both sites. At the maternity roost, we defined visitation time as any batch of detections that was <15 min apart at tower M1 because preliminary observations revealed that bats could leave and be detected by other towers for about 15 min before coming back to the roost. For the hibernaculum, we defined visitation time with a 30 min threshold (Gallant and Broders 2015) at tower H1, but our analyses revealed no differences in interpretation whether we used a 15, 30, or 45 min threshold. We used generalized linear models to compare visitation time per night between site and sexes while allowing for different standard deviations by groups.

For both return rate and visitation time analyses, our telemetry configuration did not allow differentiating when bats were inside the hibernaculum or maternity roost. Rather, it confirmed whether bats were within 500 m of each site.

### Distribution of bat activity.

To understand bat behavior away from the maternity roost and the swarming site, we examined the directionality of their activity. We calculated the bearing from the maternity roost/swarming site to each tower and used the number of visits per tower as a proxy of bat activity. We defined a visit to a tower as any batch of detections that was <2 min apart (series of detections without a gap that exceeded 2 min). We examined the activity level in relation to the bearing from the maternity roost/swarming site. We used circular statistics from the “circular package” (Agostinelli and Lund 2022) and performed a Rayleigh test to evaluate if activity was distributed uniformly around each site (Landler et al. 2018). The Rayleigh test measures data dispersion around a central point by looking at the mean resultant length, a value between 0 and 1 referring to the degree of concentration around a point (Cremers and Klugkist 2018). To account for unequal numbers of visits among bats, we performed 1,000 bootstrap resamples, using the smallest number of visits recorded at each site (18 visits at the maternity roost and 1 visit at the swarming site) to evaluate grouped activity distribution. The smallest number of visits was chosen to ensure that all bats were considered—23 bats for the maternity roost and 37 bats for the swarming site. For the swarming site analysis, we also tested bootstrap resampling with 6 and 16 visits. Although these scenarios included only 19 and 15 bats, respectively, the results were consistent with those obtained using just 1 visit per individual. To maximize the number of individuals considered, we used 1 visit per bat in the final analysis.

### Habitat preferences.

We evaluated habitat preferences by calculating the relationship between the number of detections at each antenna and the proportion of habitat cover within the range of each antenna. The number of detections by antenna was used as a proxy for bat activity. Automated telemetry does not provide exact positions of bats, but the distribution of detections among antennas is proportional to the use of the area in the surroundings (antennas with the most detections indicate where bats spent the most time), providing a relative index of habitat preferences. We excluded towers M1 and H1 in this analysis, as their proximity to the maternity roost or hibernaculum would bias activity estimates. We defined the detection range of each antenna by dividing a 500 m circular buffer around towers in 4 quadrants centered on each antenna (Figs 1 and 2).

We characterized the habitat within the detection range of each antenna by extracting 12 habitat features that influence bat activity (Table 1). We used the 2019 Québec Land Use Classification to extract habitat cover and edges at each antenna (10 m resolution; ministère de l’Environnement et de la Lutte contre les changements climatiques 2022). We used the 2015 Québec Forest Survey data to characterize forest age and harvest activity (ministère des Forêts, de la Faune et des Parcs 2022a, 2022b). The Québec Forest Survey assesses age by coring dominant trees in the field and using models to estimate stand age, which is then grouped into age classes (ministère des Forêts, de la Faune et des Parcs 2022c). Using QGIS software (QGIS.org 2022), we pooled forest stands into 2 categories: young growth (<80 yr old) and old growth (>80 yr old). We calculated the minimum Euclidian distance between towers and the maternity roost/hibernaculum to account for the spatial attraction to those sites. For the maternity roost, we also calculated the minimum Euclidian distance between towers and the Désert River, a major landscape feature in the study area (Fig. 1).

Habitat metrics at each tower are presented at Supplementary Data SD4 and SD5. Landscape around the maternity roost mostly consisted of forest (mostly young growth), agriculture comprising mostly perennial crops, and harvested forest. The harvested forest surrounding the maternity roost primarily comprised areas subjected to cutting with regeneration and soil protection, along with regular selective cutting conducted within the past 4 yr. Landscape at towers around the swarming site was dominated by a balanced mix of old and young growth forest, water (mostly lakes), wetlands, and sparse residential areas.

We evaluated the influence of habitat on bat activity by developing 7 candidate hypotheses (Table 3) with uncorrelated predictors (Pearson *r *< 0.6; Harrison et al. 2018). We used linear mixed effect models to account for repeated measures with random effect of bat tag ID using the “lmertest” package (Kuznetsova et al. 2017). We chose the best fit hypotheses based on the Akaike information criterion corrected for small sample size (AICc; Burnham et al. 2011) and considered all hypotheses with ΔAICc < 2 as equivalent (Burnham et al. 2011; Harrison et al. 2018). We quantified effect sizes with model coefficient estimates, evaluated significance with confidence intervals and assessed goodness of fit with pseudo-*R*^2^ (Nakagawa et al. 2017). All variables were Z-standardized before analysis.

**Table 3.** Candidate hypotheses and mechanisms to explain Little Brown Bat activity around a maternity roost (June to July 2021) and a swarming site (September 2021) in the Outaouais region, Québec, Canada. Linear mixed effect model structures are presented for each hypothesis. See Table 1 for predicted effect of each habitat feature.

Hypotheses	Hypothesized mechanism	Model structure
**Maternity roost**		
**Base model**	Bat activity is concentrated close to the residence and the river (potential principal commuting and foraging area)	Dist. Maternity + Dist. River + (1|TagID)
**Habitat cover**	Bat activity is positively influenced by the amount of foraging habitat	%Young Forest + %Old Forest + %Water + %Wetlands +%Harvesting +Dist. Maternity + Dist. River + (1|TagID)
**Water edges**	Bat activity is positively influenced by the amount of water edge	Water edge density + Dist. Maternity + Dist. River + (1|TagID)
**Forest edges**	Bat activity is positively influenced by the amount of forest edge	Forest edge density + Dist. Maternity + Dist. River + (1|TagID)
**Anthropogenic**	Bat activity is negatively influenced by the amount of anthropogenic habitat	%Urban + %Agriculture + Dist. Maternity + Dist. River + (1|TagID)
**Null model**	Bat activity is not influenced by habitat features	1 + (1|TagID)
**Swarming site**		
**Base model**	Bat activity is concentrated close to the swarming site	Dist. Hibernaculum + (1|TagID)
**Habitat cover**	Bat activity is positively influenced by the amount of foraging habitat	%Young Forest + %Old Forest+ %Water + %Wetlands +%Harvesting + Dist. Hibernaculum + (1|TagID)
**Water edges**	Bat activity is positively influenced by the amount of water edge	Water edge density + Dist. Hibernaculum + (1|TagID)
**Forest edges**	Bat activity is positively influenced by the amount of forest edge	Forest edge density + Dist. Hibernaculum + (1|TagID)
**Anthropogenic**	Bat activity is negatively influenced by the amount of anthropogenic habitat	%Urban + %Agriculture + Dist. Hibernaculum + (1|TagID)
**Null model**	Bat activity is not influenced by habitat features	1 + (1|TagID)

## Results

### Capture and telemetry data.

#### Maternity roost

At the maternity roost, we tagged 23 lactating females (body mass = 9.31 ± 1.38 g, forearm length 39.78 ± 1.65 mm) and collected 377,023 detections from the towers over 18 d and nights in June to July 2021. We estimated that bats kept their tags on average 5 nights (range 2-8 nights). Most tags fell off in the roost, leading to constant detection power at tower M1 and a precise estimation of the number of nights most bats kept their tag (active nights). Most females spent their days in or near the maternity roost (tower M1), but we did detect 3 bats that each spent at least 1 d roosting at other locations near towers M2, M6, and M7 (Fig. 1). Using manual telemetry, we detected active flying females as far as 5 km from the maternity roost.

#### Swarming site

At the swarming site, we tagged 39 adults (24 males, body mass = 8.73 ± 1.22 g, forearm length 38.19 ± 2.02 mm; 15 females, body mass = 8.00 ± 0.81 g, forearm length 39.08 ± 1.45 mm). No females banded at the maternity roost were recaptured at the swarming site. A total of 260,582 detections were collected from towers over 28 d and nights in September 2021. One atypical male spent over 25 nights and days at tower H8 (away from the hibernaculum; Fig. 2), representing >64% of all detections. We excluded this male from our analyses as the atypical behavior and overwhelming number of detections had a disproportionate influence.

Of the 91,575 remaining detections, 85,392 were male (93%), compared with just 6,183 for females (2 sample test of proportion, χ^2^ = 59.56, *P *< 0.0001). The number of detections for males averaged 3,172 detections per individual (median = 927, range 23 to 18,924) and we estimated that they kept their tags on average 14 nights (range 1 to 27 nights). Of the 3 males that kept their tag the longest (27 nights), 2 were detected almost every night of the study period, while 1 was only detected on 2 nights.

For females, the number of detections averaged 412 per individual (median = 334, range 25 to 1071) and we estimated they kept their tags on average 6 nights (range 1 to 20 nights). The 2 females that kept their tag for the longest period of time (18 and 20 nights) were only detected 2 to 4 nights during this period and were out of detection range the rest of the time. No tags fell off in the detection range of a tower, and we conservatively estimated the time a tag fell as the last detection for each bat, although it is likely that some bats simply left the study area.

During swarming, day roost locations were generally unknown with the exception of 8 males detected roosting near towers at least for a day, 3 of which were at tower H1 close to the hibernaculum for at least a day. Using manual telemetry, we also detected a male and a female day-roosting, for a day only, at ∼10 km and ∼8.4 km away from the hibernaculum, respectively. We did not characterize these roosts because they were located on private property. Females were not detected during the day at any of the towers, which covered a radius of approximately 5 km from the swarming site. At night, the farthest detections we had using manual telemetry were a male and a female detected over a lake almost 13 km east of the cave. No bats were detected at the 2 towers placed at other hibernacula.

## Return rate and visitation time

### Maternity roost

**Fig. 3.**
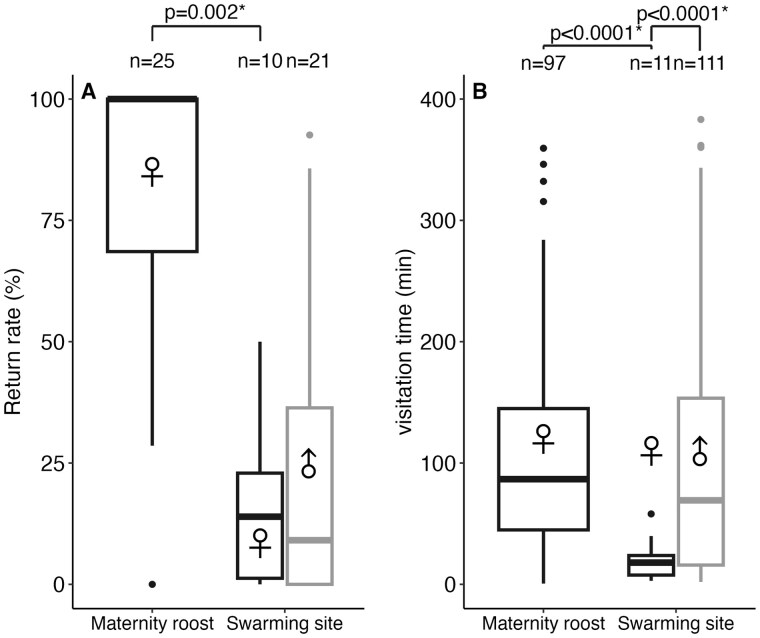
Little Brown Bat return rates and visitation behavior by sexes at a maternity roost (June-July 2021) and a swarming site (September 2021) in the Outaouais region, Québec, Canada. (A) Percent of active nights with at least 1 return to the site and the sample size (*n* = number of tagged bats). (B) Nightly visitation time (minutes) at the site and the number of nights with at least 1 visit to the site (*n* = number of bat-nights). Boxplots present median (thickest line), interquartile range (box) and most extreme data points (whiskers).

All but one female returned to the maternity roost at least once during the study period, and they returned on 81 ± 29% of nights (Fig. 3A). When they did return to the maternity roost at night, females stayed for 105 ± 84 min per night (Fig. 3B) with an average of 3 returns per night (max = 8 returns) of 34 ± 44 min.

### Swarming site

Seventy-one percent of males returned to the swarming site at least once and they return 24 ± 30% of nights there. When males did return, they remained at the swarming site 98.4 ± 96.0 min per night with an average of 2 returns per night (max = 5) averaging 50 ± 71 min per visit.

Seventy percent of females returned at least once to the cave during the study period, similar to the observed return rate for males (2 sample test = χ^2^ < 0.001, *P *> 0.99). Females returned 16 ± 16% of nights to the swarming site, again similar to males (β  =  0.55, SE = 1.00, *P *= 0.59; Fig. 2A). However, when compared to the maternity roost, female return rate at the swarming site was about 5 times less (β = −3.12, SE = 1.02, *P *= 0.002; Fig. 3A). When they did return to the swarming site, females stayed only 19.7 ± 16.7 min per night and averaged 2 returns per night (max = 4 returns) of 10 ± 12 min, which is less than males (mean difference = 78.7 min, SE = 10.4 min, *P *< 0.001; Fig. 3B). Females spent significantly less time per night around the swarming site than around the maternity roost in summer (mean difference = 85.5 ± 9.9 [SE] min, *P *< 0.001; Fig. 3B). Because bats inside the cave were not detectable, 2 returns at the swarming site might represent a single visit: bats were detected once going into the cave and then a second time as they emerged. Consequently, visitation time at the swarming site is likely underestimated.

Although no difference in return rate was observed between males and females, we note that 4 males did return to the swarming site on ≥ 12 nights (range 12 to 25 nights) during the study period. In contrast, the 1 female that was most frequently detected at the swarming site was detected only 3 nights at tower H1 out of the 18 nights she was tagged. These results suggest a difference between male and female return behavior, however, as we lost track of females early during the study period, this difference is not highlighted in the statistical analyses.

## Distribution of bat activity

### Maternity roost

**Fig. 4.**
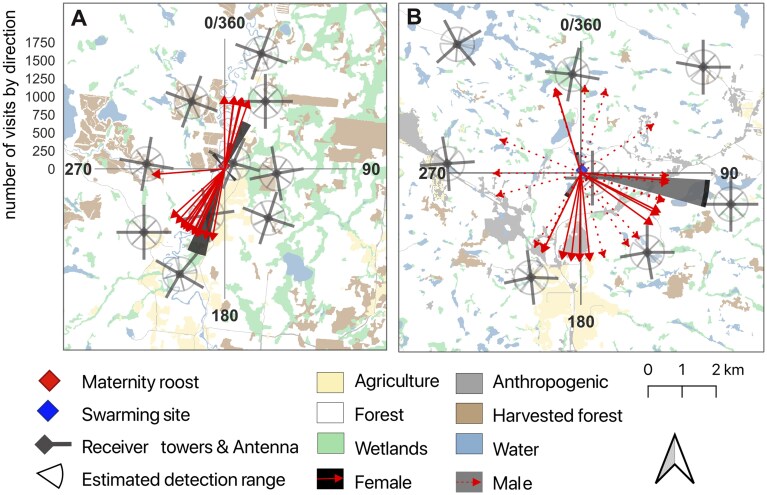
Distribution of Little Brown Bat activity around (A) a maternity roost (June to July 2021) and (B) a swarming site (September 2021) in the Outaouais region, Québec, Canada. The center of each circular plot is the maternity roost/swarming site, bars centered in the circular plots represent the absolute number of bat visits by receiver towers according to the bearing from the maternity roost/swarming site. The arrows are the mean vectors (mean directions) for individual bats.

Bat activity was not uniformly distributed around either site. At the maternity roost, combined activity from all females was biomodally distributed to the south and north of the roost. The bootstrap analysis yielded a mean resultant length (degree of concentration around a point) of 0.332 [95% CI: 0.330 to 0.333], and all but 1 bootstrap iteration was significant (*P *< 0.05), indicating non-uniform bat activity around the roost. All but 1 individual bat consistently exploited habitats in either the northern or southern direction of the maternity roost (Fig. 4A; *P *< 0.0001; Supplementary Data SD2).

### Swarming site

At the swarming site, bat activity (males and females combined) was concentrated to the south-east (mean bearing of activity = 123° [95% CI: 120 to 124]) and the bootstrap analysis yielded a mean resultant length (degree of concentration around a point) of 0.180 (95% CI: 0.172 to 0.183). All but 4 bootstrap iterations were significant (*P *< 0.05), indicating non-uniform bat activity around the swarming site. However, we observed no clear pattern when looking at individual bat directionality (Fig. 4B): 40% had a uniformly distributed activity pattern (6 of 14 females and 9 of 23 males; *P *> 0.05), while the others concentrated their activity in 1 direction, but no particular direction was consistent among bats (*P *< 0.05; Supplementary Data SD3). Still, most bats had a low number of detections at the swarming site, therefore we might not be capturing all the activity distribution in the surroundings.

## Habitat preferences

### Maternity roost

**Fig. 5.**
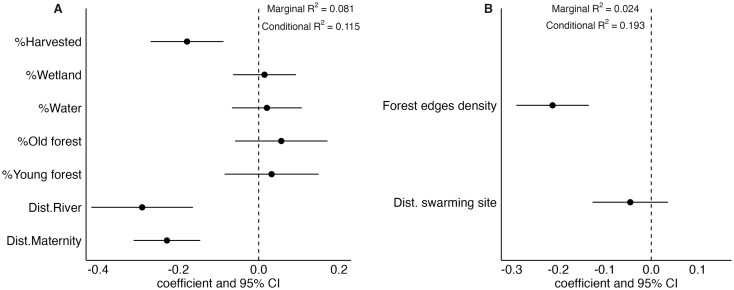
Habitat features influencing Little Brown Bat activity in the Outaouais region, Québec, Canada. Linear mixed effect model coefficient estimates (points) and 95% confidence intervals on coefficient estimates (bars; 95% CI) for all habitat features included in the best supported hypotheses for (A) female Little Brown Bat activity around a maternity roost (June to July 2021) and (B) combined male and female bat activity around a swarming site (September 2021). Habitat features are considered to have negative effects on bat activity when the 95% confidence intervals are entirely below zero, and positive effects when entirely above zero.

Habitat features partially explained bat activity at both sites. Bat activity around the maternity roost supported the *habitat cover* hypothesis (Table 4). Bat activity decreased with distance from the river (ß = −0.290, SE = 0.064, *t*_1653_ = −4.50, 95% CI = [−0.417, −0.164]) and distance from the maternity roost (ß = −0.229, SE = 0.042, *t*_1640_ = −5.40, 95% CI= [−0.312, −0.146]; Fig. 5A). Less activity occurred in areas with high harvested forest cover (ß = –0.178, SE = -0.046, *t*_1626_ = −3.87, 95% CI = [−0.269, −0.088]; Fig. 4A). However, while habitat partially explained activity patterns, the effect was small as habitat only explained 8% of the variation in activity patterns (marginal *R*^2^ = 0.081; Table 4). Individual preferences exerted a stronger influence on the level of activity according to conditional *R*^2^ (Table 4).

**Table 4.** Model summaries explaining Little Brown Bat activity around a maternity roost (June to July 2021) and a swarming site (September 2021) in the Outaouais region, Québec, Canada. Candidate models are ranked by AICc, ΔAICc, and AICc weight (ω). Pseudo-marginal and pseudo-conditional *R*^2^ also presented for each hypothesis. Only hypotheses with ΔAICc < 2, highlighted in light grey, were considered for subsequent analyses. Candidate model descriptions and linear mixed effect model structure are presented in Table 1.

Hypothesis	AICc	ΔAICc	ω	Marginal *R* ^2^	Conditional *R* ^2^
**Maternity roost**					
**Habitat cover**	5,770.69	0.00	0.95	0.081	0.115
**Anthropogenic**	5,776.70	6.01	0.05	0.074	0.113
**Forest edges**	5,785.46	14.77	0.00	0.068	0.108
**Base**	5,787.79	17.09	0.00	0.065	0.104
**Water edges**	5,788.02	17.33	0.00	0.066	0.106
**Null**	5,898.60	127.90	0.00	0.000	0.038
**Swarming site**					
**Forest edges**	4,302.15	0.00	0.98	0.024	0.193
**Anthropogenic**	4,310.07	7.92	0.02	0.020	0.192
**Habitat cover**	4,324.07	21.92	0.00	0.014	0.180
**Base**	4,328.27	26.12	0.00	0.003	0.187
**Water edges**	4,328.73	26.58	0.00	0.004	0.186
**Null**	4,330.28	28.12	0.00	0.000	0.181

### Swarming site

Bat activity around the swarming site supported the *forest edge* hypothesis, although with a direction contrary to our prediction (Table 4). Bat activity decreased with increasing forest edge density (ß = −0.212, SE = 0.040, *t*_1267_ =−5.34, 95% CI = [−0.290, −0.134]; Fig. 5B). However, contrary to the maternity roost, the distance to the hibernaculum had no effect on bat activity (Fig. 5B). We did not separate males and females for the habitat preference analysis at the hibernaculum because more than 92% of the detections from towers (other than tower H1) are from males. Therefore, the habitat preference analysis is highly male biased. Habitat explained only 2.4% of variation in bat activity at the swarming site (Table 4).

## Discussion

Central place behavior imposes spatial constraints on animals, shaping their habitat use around important sites such as roosts or breeding sites (Orians and Pearson 1979). We contrasted activity patterns of the Little Brown Bat around a maternity roost in summer, during lactation, and at a hibernaculum during the swarming period in late summer. Consistent with our hypothesis, the spatial and temporal patterns of bat activity differed between the 2 sites. These variations are likely influenced by trade-offs associated with different constraints across seasons. During the summer, female activity at the central place is primarily shaped by the constraints of rearing pups, with frequent return and activity concentrated in the vicinity of the roost. In contrast, during the swarming season, central place behaviors are driven by sex-specific trade-offs between pre-hibernation fat accumulation and mating. We found that females apparently returned less often than males to the swarming site, and the lack of detections from females suggests that they may have foraged and roosted much farther away. Sex-based behavioral differences are particularly understudied during swarming, highlighting critical considerations for conservation strategies that aim to protect both sexes effectively.

## Activity at the Central place.

### Maternity roost

During lactation, female little brown bats face tradeoffs between nursing at the roost, foraging, and commuting activities (Kurta et al. 1989; Henry et al. 2002). Lactating females, compared to non-reproductive females, return to the roost once or more per night to feed pups (Henry et al. 2002; Fontaine et al. 2024), and therefore need to decrease travel distance to foraging areas (Henry et al. 2002) while increasing food intake (Anthony and Kunz 1977; Kurta et al. 1989). This behavioral shift suggests a strong spatial dependence on the roost during this period, with bats going back and forth to foraging areas and resting while nursing in the maternity roost (Henry et al. 2002). The return pattern we observed corresponds to other Little Brown Bat maternity colonies (Anthony and Kunz 1977; Henry et al. 2002; Fontaine et al. 2024). In contrast with reproductive females, male little brown bats are solitary during the summer and have no strong ties to a specific roost (Davis and Hitchcock 1965; Broders and Forbes 2004) representing the other extreme end of the central place tradeoff spectrum.

Our estimated visitation time to the roost (105.2 ± 84.0 min) was nearly identical to what was observed in lactating little brown bats by Henry et al. (2002; 102 ± 66 min). Although our methodology could not definitively determine whether a bat was inside the maternity roost or roosting somewhere very close by, the similarity in visitation times between our study and previous studies suggests that our estimated visitation time encompassed mostly resting and nursing activity in the roost rather than foraging in the surroundings.

### Swarming site

In late summer, bats are constrained to tradeoff reproductive activity—namely social interactions and mating at the hibernaculum (swarming site), and foraging to deposit fat before hibernation (McGuire et al. 2009; Gallant and Broders 2015). During swarming, however, bats generally do not roost in hibernacula (Parsons and Jones 2003; Brack 2006; McGuire et al. 2009; Gallant and Broders 2015), rather using hibernacula mostly as mating sites (Furmankiewicz 2008; but see Davis and Hitchcock 1965; Fenton 1969; and Fraser and McGuire 2023 for alternative hypotheses). To balance between mating and fat deposition, bats seem to spend a few nights at the hibernaculum, as evidenced by the 22% nightly return rate we observed (no statistical difference between 24% for males and 16% for females). Because many bats were out of detection range for days in between returns, we suggest that their activity range extended farther than our study area, likely >13 km from the hibernaculum, which was our farthest detection.

As a promiscuous species (Thomas et al. 1979), sex-biased behavioral patterns during mating are expected including that males should spend more time at the swarming site than females to maximize mating opportunities (Furmankiewicz 2008; Burns and Broders 2015). Sex-specific energetic constraints are also anticipated, females being usually more energy-depleted than males after rearing pups but also usually requiring more fat during hibernation than males (Jonasson and Willis 2011, 2012; Burns and Broders 2015). Male bias in capture at swarming sites has been observed in many species (Rivers et al. 2006; Furmankiewicz 2008; Burns and Broders 2015; Dekeukeleire et al. 2016), yet behavioral differences related to sex have rarely been quantified.

We did not detect a consistent difference in return rates between the sexes at the swarming site. We acknowledge that return rates might be biased because the exact duration of tag retention is unknown. We did lose track of females earlier in the study but there is no reason to assume that tag retention in males and females would follow the same distribution. However, the disproportionate number of detections around the swarming site and the absolute higher number of returns from males compared to females suggest that at least some males stayed closer in general and tended to return more frequently (also suggested by Furmankiewicz 2008; Burns and Broders 2015). Indeed, different males could employ different swarming strategies which might not be consistent throughout the duration of the swarming season.

Males did have longer visitation times than females, suggesting that when they do return they spend more time actively swarming than females. Our estimated visitation time might include non-swarming activity because the detection range of the H1 tower encompassed a lake and a marsh surrounded by deciduous forest. However, if this was an advantageous foraging or roosting habitat, there is no reason why females would not use it as well. Moreover, we may have underestimated the time spent at the swarming site as bats were not detected by the tower while inside the cave. Infrared cameras or other detectors could be placed inside the cave to counter this limitation, although individual activity assessment would not be possible.

Overall, low return rates at the swarming site suggest greater flexibility in habitat use in the surroundings. If bats return at least once for hibernation (Van Schaik et al. 2015), we suggest that the swarming site imposes a flexible catchment area which bats can partition for roosting and foraging activity in late summer. The low return rate observed may represent a strategy used by bats to (i) mitigate competition around hibernacula which can attract thousands of bats at a time of year when food resources are decreasing (Fenton 1969; Brack 2006; Wang et al. 2010); (ii) reach farther high-quality habitat patches to promote fat deposition before hibernation (McGuire et al. 2009; Jonasson and Willis 2011); or (iii) visit other swarming sites in the area (e.g., Fenton 1969; Brack 2006; Rivers et al. 2006), although many species seem to be faithful to 1 swarming site (Parsons and Jones 2003; Burns and Broders 2015) and we did not detect our tagged bats at the 2 other known major hibernacula in the area.

## Movement and habitat preferences around the Central place.

### Maternity roost

Maternity colonies are usually located near potential foraging areas (e.g., riparian habitat; Legros et al. 2024), likely to fulfill for the need to return regularly to the roost while having access to riparian habitats, which are considered high quality for insectivorous bats ­(Holloway and Barclay 2000; Ford et al. 2005; Fukui et al. 2006). Accordingly, bats in our study concentrated activity close to the maternity roost (∼5 km), where vegetated riparian habitat was abundant. Notably, individual bats travelled along the Désert River, either north or south of the maternity roost. Dividing into a northern and southern group might be a strategy to avoid competition and prey depletion near the roost (Hillen et al. 2009). The Désert River likely provides crucial food resources for lactating bats with low commuting costs. Corroborating the idea that habitat preference is driven by habitat quality, and not only by proximity, putatively lower-quality habitats in the vicinity, like harvested forests, were avoided. Bat activity is known to decrease within harvested patches (Deans et al. 2005; Dodd et al. 2012; but see Patriquin and Barclay 2003; Law et al. 2016), which supports the negative effect of harvested cover found in our study.

Our study highlights the application of automated telemetry for the broad assessment of habitat use. Our towers were not located randomly, but rather were placed uniformly to maximize coverage of our study area. Automated detections do not provide precise location estimates, but provided an index of habitat preferences that was able to detect preference for riparian areas, avoidance of forest harvested areas, and variation in individual movement patterns (north and south of the roost). Studies focused on more specific or detailed habitat preferences may require more labor-intensive manual tracking, but automated telemetry is clearly effective for many questions.

### Swarming site

Individual bats focused their activity in different directions from the hibernaculum and thus exploited different habitat patches surrounding the swarming site, but we did not observe any strong habitat preferences. Overall, we recorded fewer detections at the swarming site in September compared to the maternity colony in summer and we found bats farther than the area covered by our automated telemetry network. Therefore, our methods provided good information about activity patterns in the immediate vicinity of the swarming site, but a comprehensive assessment of activity away from the swarming site would require a broader spatial scale than the tower network we deployed.

It is difficult to draw any clear conclusions about what kind of habitat bats exploited for roosting and foraging during the swarming period because we detected only a fragment of their overall activity. For instance, there is no reason that bats would not use riparian areas as foraging habitat or travel corridors during swarming. However, our towers covered only a small proportion of lakes and rivers in the area (Fig. 2).

Our analysis provided weak evidence suggesting that swarming bats avoid areas with high forest edge density. While forest edges are usually good foraging and commuting habitat for bats (Crampton and Barclay 1998; Patriquin and Barclay 2003), the negative effect of forest edge density could illustrate a preference for forest interior, which may offer adequate roosting habitat (Crampton and Barclay 1998; Grindal and Brigham 1999). Indeed, bats may use torpor to conserve energy in late summer (McGuire et al. 2016) and we suspect that constant and stable night detections made by some towers could be associated with either night-time torpor bouts or roosting.

Laflèche Cave is one of the few hibernacula in Québec where the bat population has increased following initial declines caused by white-nose syndrome. Across the province, hibernaculum population size prior to the arrival of white-nose syndrome in Québec correlated positively with forest edge density and negatively with anthropogenic habitat in the surroundings (Legros et al. 2024). The area around Lafleche Cave is mostly composed of natural forests, lakes, wetlands, and minimal human activity. Consequently, this hibernaculum might already provide a suitable habitat matrix for bats, especially at lower density following white-nose syndrome decline, a possible explanation of the low-explanatory power of the habitat preference model.

## Sex-specific central place behavior.

Our study highlights seasonal variation in sex-specific constraints and tradeoffs, with different central-place behavior trends for male and female bats during summer and mating seasons. In summer females are tied to their maternity roosts and have high energetic demands associated with rearing pups, while males are not bound to a specific roost. Although it has been suggested that males may be excluded from high-quality habitat during the summer (Encarnação et al. 2005; Senior et al. 2005), they remain less restricted in their movements between foraging habitat patches and can use daily torpor bouts to manage their energy budget in poor foraging conditions (e.g., Grinevitch et al. 1995). The localized movement constraints for reproductive females highlight the importance of ensuring high-quality habitats around maternity roosts.

During the mating season in late summer, both sexes are tied to the swarming site, but the costs and benefits differ. Females must deposit relatively more fat for hibernation than males, and males may benefit more from spending more time at the mating site than do females (Kunz et al. 1998). Females may therefore prioritize fattening over copulation because they are not likely to increase their fitness by multiple copulations (Burns and Broders 2015), which may lead to spending more time at high-quality foraging habitats away from the swarming site. However, because little is known about female activity patterns during the swarming period, and many questions about the ecology of female bats remain during this period.

Conversely, males might allocate more active time at the swarming site to maximize mating opportunities and increase fitness (Burns and Broders 2015), which may impose a tradeoff on pre-hibernation fattening, constraining males to remain near the swarming site and precluding the opportunity to spend time at high-quality foraging sites distant from the swarming site. However, the wide variation in return rates among males (e.g., the male that stayed around tower H8 throughout the study period) led us to believe that different intrasexual strategies exist for balancing mating and foraging.

Bats were found day roosting more than 10 km away from the swarming site, and some may be roosting farther. More than 30% of males were found day roosting near a tower within a radius of about 5 km, which is consistent with male little brown bats tracked at a swarming site in Nova Scotia (Lowe 2012) and male Indiana bats (*M. sodalis*; Perry et al. 2016). However, only one female was detected during the day (8 km away from the swarming site). Because we detected few night movements from females in general, we could expect that they were roosting and foraging away from the array of towers most of the time (covering a radius of about 5 km around the cave). This highlights the need to investigate sex-specific and individual variations at swarming sites, particularly in a conservation context. If male activity (foraging and roosting) is more concentrated near the swarming site compared to females, management strategies focused on a small protection radius around the hibernaculum may not adequately protect females. For instance, according to the Bat Conservation Strategy for Forest Service-Managed Lands of the Eastern United States (US Forest Service and US Fish and Wildlife Service 2024), Laflèche Cave would be considered a *Moderate Abundance ­Hibernaculum*. Thus, a 3.2 km buffer of protection would be applied around the cave—a buffer that might only protect some males and not females.

## Limitations and improvements.

Automated telemetry can provide new opportunities for research on small, cryptic, and fast-moving animals, allowing near-continuous and simultaneous monitoring of many individuals. Automated telemetry is not an equivalent substitute for conventional tracking and detailed habitat selection studies but necessitates and allows more and different questions to be addressed. For example, automated telemetry allows the simultaneous tracking of many individuals, presenting a tradeoff in the spatial precision of location estimates and the sample size available to test hypotheses and make population-level inferences. Telemetry studies, whether manual tracking or tower-based automated telemetry, frequently lose track of focal animals. Our tracking networks did not capture all bat movements and activity around the maternity roost and the swarming site. We lost track of many females at the swarming site, and their activity areas remain unknown. Automated telemetry can be considered in a probabilistic context, and the absence of detections can be informative. Although we do not know where females spent most of their time during swarming, we do know that it was not near the swarming site, and unlikely within a 5 km radius of the hibernaculum.

To improve our design, we recommend, when possible, to increase the number of towers, balancing the coverage within the study area with the spatial extent of the study. Towers can be placed in high density over smaller spatial scale, or at lower density over a broad area as dictated by the specific research questions of the study. Within the study area tower locations should be informed by the research question, and covering the greatest diversity of habitat types possible. Covering greater habitat diversity is generally likely to be beneficial, but specific a priori information may dictate disproportionate coverage of some habitats. For example, in our study we a priori expected bat activity along the main riparian corridor and ensured that our towers provided coverage of this habitat.

Future studies can overcome some of the limitations of automated telemetry networks by employing complementary techniques. For example, our automated telemetry tower could not detect bats inside the cave during swarming, but placing an additional telemetry receiver inside the cave, or installing trail cameras or infrared cameras at the entrance of the cave could help define peak entry and emergence periods of tagged or untagged bats. Similar approaches could be taken at the maternity roost, offering greater insight into the timing and return rates associated with each phase of the annual cycle. Furthermore, deploying acoustic detectors in favorable habitats such as riparian areas could help document habitat use to complement tracking data, especially in areas where towers cannot be deployed. Finally, future studies at maternity roosts and hibernacula in different landscapes would complement our findings, as habitat selection may differ even within a geographic region (Perry et al. 2016).

## Implications

The use of maternity roosts embodies the typical consideration of a central place because bats exhibit high spatial dependence, commuting within a ∼5 km radius to nearby common habitat patches (Orians and Pearson 1979; Rosenberg and McKelvey 1999). During lactation, this behavior might be facilitated by high insect density, providing high-quality food sources and reducing competition near the roost (Henry et al. 2002). Protecting critical habitats such as riparian corridors near maternity roosts is thus important. The Bat Conservation Strategy in the United States does prescribe a protection buffer of 1.1 km around maternity roosts, with the purpose of protecting secondary roosts, which are important to account for roost-switching behavior in bats roosting in trees (e.g., Slough and Jung 2020). However, management plans should also consider protection buffers that encompass critical foraging habitat and are adapted for maternity roosts in buildings, which are common in northern regions and can sometimes host larger numbers of bats.

Hibernacula function more as “focal places” during swarming in late summer, attracting bats for mating and ultimately hibernation. Bats may travel from the hibernaculum up to ∼13 to 20 km during this period for foraging and roosting (Parsons and Jones 2003; Dekeukeleire et al. 2016), highlighting the importance of a connected matrix of natural habitats within the catchment area to facilitate movement, minimize competition, and support pre-hibernation fattening. Habitat requirements during the maternity season are relatively consistent from 1 night to the next (nearby high-quality foraging habitat to support lactation and pup rearing). Conversely, habitat requirements may vary night-to-night and between males and females during the swarming season because bats may prioritize fattening and mating on different nights. Some males tended to stay closer to the swarming site, while females rarely made use of habitat in the immediate surroundings. Thus, it is important to consider the matrix of habitat availability at different scales. Conservation strategies with protective buffers around hibernacula should be refined to account for both males and females, and developed in areas where they do not yet exist. However, knowledge of Little Brown Bat (or most species for that matter) habitat preferences and behavior during autumn remains limited (Fraser and McGuire 2023; Frick et al. 2023), warranting further research combining complementary techniques during this critical period of the annual cycle.

## Supplementary Material

gyag001_Supplementary_Data

## Data Availability

The clean datasets of bat detections per receiver tower and per night are available in the Open Science Framework repository at [https://osf.io/f2dhr/? view_only=fed7eb60e2cd40efb2cea5cd6e8e34fd]

## Supplementary data

**Supplementary Data SD1**. Methods of estimation of the detection range of the Receiver Towers.

**Supplementary Data SD2**. Directionality and rayleigh test results for individual female little brown bats at the maternity roost.

**Supplementary Data SD3**. Directionality and rayleigh test results for individual little brown bats at the swarming site.

**Supplementary Data SD4**. Habitat feature covered by receiver towers around the maternity roost.

**Supplementary Data SD5**. Habitat feature covered by receiver towers around the swarming site.
