# Supplementary material for: Contrasting spatial and temporal activity patterns of little brown bats (Myotis lucifugus) at maternity roosts and swarming sites
Source: J Mammal. 2026 Feb 5;107(2):334–47. doi: 10.1093/jmammal/gyag001 (PMC13035269; doi:10.1093/jmammal/gyag001)
Supplement: gyag001_Supplementary_Data [file gyag001_supplementary_data.docx]

# SUPPLEMENTARY DATA

*SD1: Methods of estimation of the detection range of the receiver towers*

We evaluated the detection range of the towers in the study areas using different methods. We waved a test tag for three minutes at known locations around towers (every ~250 m till about 1500 m from the tower) using one of the following methods: tag mounted on a wooden stick (~5 m into the air); tag attached to a 50 ml saltwater tube mounted on a wooden stick (~5 m into the air) and tag attached to a 50 ml saltwater tube hanged to a drone (DJI Phantom 4, TS2 Space, Poland) with 100 cm rope (fly with constant rotation at five meters, 15 m and 30 m into the air). We used a saltwater tube to mimic the body of the animal. We did not find any major differences in detection range evaluated with the different methods and height. Depending on accessibility, we tested the detection range of at least one antenna for each tower. We tested the towers at 182 locations at the maternity roost and at 108 locations at the hibernaculum. Towers detected test tag up to 3060 m away, but generally much closer with a median detection range of 335.4 m and a means of 462.7 m (Figure S2.1). We acknowledge that it is impossible to accurately mimic the effect of the animals with a tag, and that those tests only give an approximation of the detection range of our towers.

We also estimated the detection range of towers using simultaneous detections of bats between towers. When simultaneous detections occurred, it was from towers separated on average by 2072 m (median= 1916 m, max= 6096 m), which suggest a detection range of about 1036 m by towers. However, simultaneous detections represented 1.06% of total detections, suggesting that they were rare events. We estimated that the effective detection range of our towers was between 500 m and 1000 m, and consider 500 m as a conservative detection range (Crewe et al., 2019). We acknowledge that detection range might vary among towers depending on the landscape and line of sight (Crewe et al., 2019; Taylor et al., 2017).


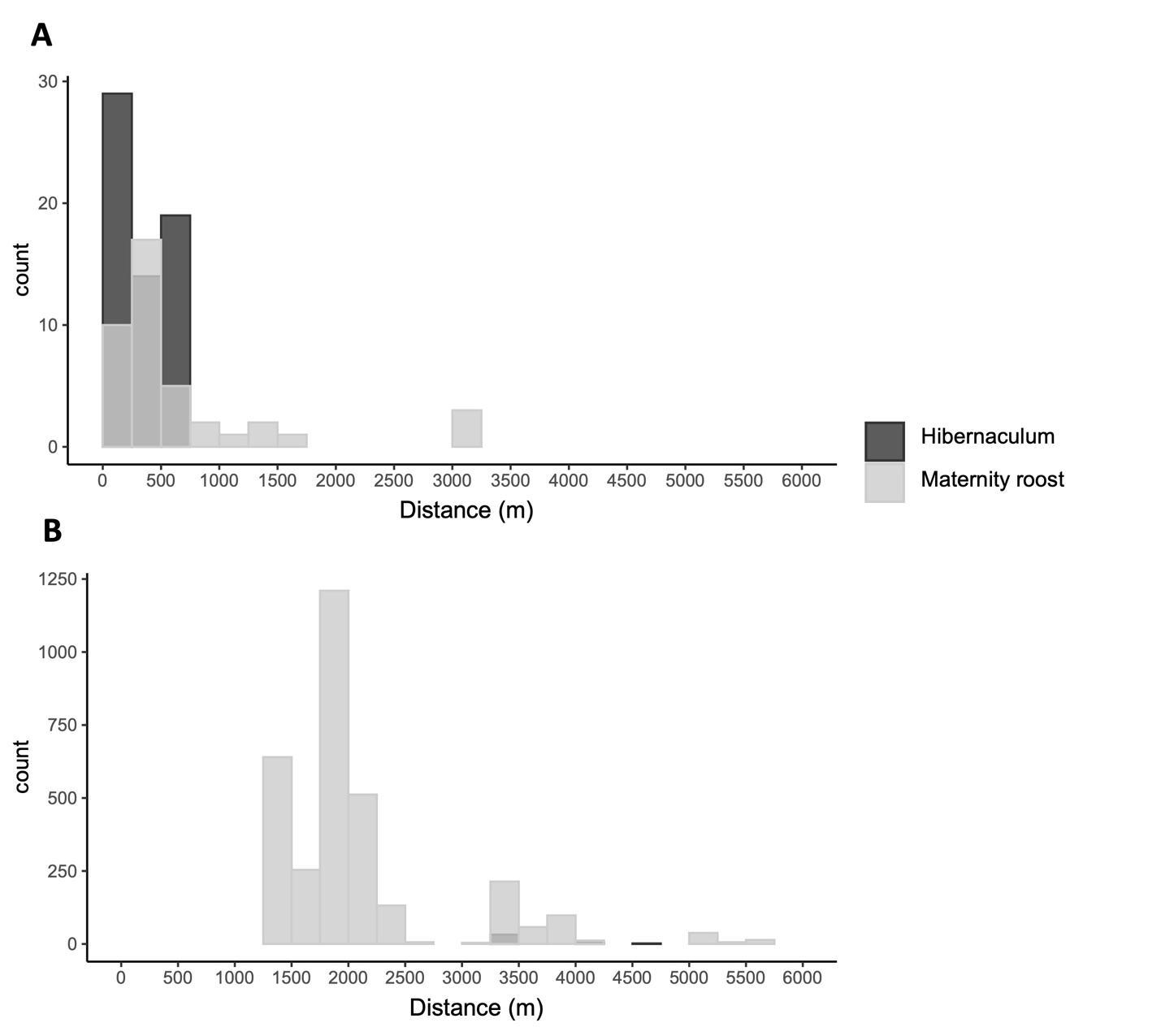


**Fig. SD1**. Evaluation of the detection ranges of the automated receiver towers at the maternity roost and hibernaculum A) Distance between towers and location of test tags detected ; B) Distance between towers when simultaneous detection of a bat happened.

*SD2: Directionality and rayleigh test results for individual female little brown bats at the maternity roost*

**Table SD2**. Mean direction of little brown bat activity surrounding a maternity roost according to the number of visits to a tower and it’s bearing from the roost. Result from Rayleigh test of uniformity, mean resultant length and p values.

| Tag ID | Sexe | Mean direction | Mean resultant length | P-Value |
| --- | --- | --- | --- | --- |
| 465 | F | 190.078 | 0.748 | <0.001 |
| 442 | F | 7.312 | 0.899 | <0.001 |
| 463 | F | 203.066 | 0.617 | <0.001 |
| 464 | F | 227.692 | 0.462 | 0.002 |
| 480 | F | 216.458 | 0.666 | <0.001 |
| 481 | F | 210.889 | 0.750 | <0.001 |
| 482 | F | 13.110 | 0.714 | <0.001 |
| 483 | F | 195.889 | 0.892 | <0.001 |
| 484 | F | 211.880 | 0.463 | <0.001 |
| 485 | F | 214.415 | 0.580 | <0.001 |
| 486 | F | 212.164 | 0.741 | <0.001 |
| 488 | F | 14.168 | 0.852 | <0.001 |
| 489 | F | 216.939 | 0.769 | <0.001 |
| 490 | F | 203.472 | 0.822 | <0.001 |
| 491 | F | 19.307 | 0.520 | <0.001 |
| 492 | F | 221.901 | 0.804 | <0.001 |
| 493 | F | 7.781 | 0.742 | <0.001 |
| 494 | F | 359.582 | 0.362 | <0.001 |
| 495 | F | 265.516 | 0.203 | 0.193 |
| 496 | F | 201.805 | 0.938 | <0.001 |
| 497 | F | 206.613 | 0.570 | <0.001 |
| 498 | F | 202.286 | 0.777 | <0.001 |
| 499 | F | 205.964 | 0.871 | <0.001 |

| Tag ID | Sexe | Mean direction | Mean resultant length | P-Value |
| --- | --- | --- | --- | --- |
| 437 | F | 116.310 | 0.931 | 0.019 |
| 438 | M | 269.858 | 1.000 | 0.137 |
| 439 | M | 146.991 | 0.747 | <0.001 |
| 440 | F | 192.407 | 0.885 | <0.001 |
| 441 | F | 94.864 | 1.000 | 0.033 |
| 443 | F | 205.883 | 1.000 | 0.512 |
| 444 | M | 94.864 | 1.000 | <0.001 |
| 445 | M | 255.005 | 0.655 | 0.116 |
| 446 | M | 91.665 | 0.925 | <0.001 |
| 447 | F | 343.244 | 1.000 | 0.512 |
| 448 | M | 164.683 | 0.835 | <0.001 |
| 449 | F | 343.244 | 1.000 | <0.001 |
| 450 | M | 138.642 | 0.633 | <0.001 |
| 451 | M | 206.886 | 0.942 | <0.001 |
| 452 | F | 125.854 | 0.678 | <0.001 |
| 453 | F | 343.244 | 1.000 | 0.033 |
| 454 | M | 56.189 | 0.703 | 0.140 |
| 455 | M | 205.883 | 1.000 | 0.137 |
| 456 | M | 2.700 | 0.698 | 0.144 |
| 457 | M | 342.611 | 0.992 | <0.001 |
| 458 | M | 16.654 | 0.608 | <0.001 |
| 459 | M | 212.055 | 0.788 | 0.004 |
| 460 | F | 205.883 | 1.000 | 0.512 |
| 461 | M | 343.244 | 1.000 | 0.001 |
| 462 | M | 96.819 | 0.955 | <0.001 |
| 468 | M | 95.181 | 0.992 | <0.001 |
| 469 | F | 186.419 | 0.700 | 0.015 |
| 470 | M | 94.864 | 1.000 | 0.137 |
| 471 | M | 343.244 | 1.000 | 0.512 |
| 472 | F | 181.003 | 0.737 | 0.031 |
| 474 | M | 109.092 | 0.812 | <0.001 |
| 475 | F | 118.310 | 0.303 | 0.133 |
| 476 | M | 107.921 | 0.098 | 0.958 |
| 477 | M | 94.864 | 1.000 | <0.001 |
| 478 | F | 116.310 | 0.931 | 0.193 |
| 479 | F | 174.182 | 0.708 | 0.075 |
| 513 | M | 292.601 | 0.826 | 0.128 |

*SD3: Directionality and rayleigh test results for individual little brown bats at the swarming site*

**Table SD3.** Mean direction of little brown bat activity surrounding a swarming site according to the number of visit to a tower and it’s bearing from the swarming site. Result from Rayleigh test of uniformity, mean resultant length and p values.

*SD4: Habitat feature covered by receiver towers around the maternity roost*

**Table SD4.** Habitat metrics for each antenna of the ten receiver towers installed around a little brown bat maternity roost (June-July 2021) in the Outaouais region, Québec, Canada. Habitat metrics were extracted from 2019 Québec Land Use Classification (10 m resolution; ministère de l’Environnement et de la Lutte contre les changements climatiques, 2022), forest age and harvested forest cover (% harvested) metrics were derived from 2015 Québec Forest Survey data (ministère des Forêts, de la Faune et des Parcs, 2022a, 2022b).

| Tower | Antenna | % Young forest | % Old forest | % Water | % Wetlands | % Urban | % Harvested | % Agriculture | Water edges  (m/ha) | Forest edges  (m/ha) | Distance to roost (m) | Distance to river (m) |
| --- | --- | --- | --- | --- | --- | --- | --- | --- | --- | --- | --- | --- |
| 1 | 1 | 32.09 | 60.46 | 5.66 | 0.00 | 0.00 | 0.56 | 0.00 | 60.14 | 67.28 | 0 | 380.00 |
|  | 2 | 39.58 | 49.77 | 0.00 | 0.00 | 0.20 | 0.00 | 11.30 | 0.00 | 31.06 |  |  |
|  | 3 | 11.71 | 8.91 | 0.00 | 33.88 | 2.80 | 0.00 | 37.54 | 0.00 | 96.79 |  |  |
|  | 4 | 42.64 | 17.22 | 1.58 | 5.40 | 1.63 | 0.00 | 18.81 | 14.27 | 148.32 |  |  |
| 2 | 1 | 90.77 | 0.00 | 0.41 | 0.00 | 5.35 | 2.95 | 0.00 | 8.15 | 68.74 | 3325.00 | 314.00 |
|  | 2 | 67.21 | 0.00 | 0.00 | 8.11 | 22.79 | 0.00 | 0.00 | 0.00 | 114.74 |  |  |
|  | 3 | 74.71 | 3.26 | 3.87 | 11.21 | 0.25 | 5.35 | 0.00 | 36.70 | 126.91 |  |  |
|  | 4 | 86.37 | 0.00 | 10.48 | 2.04 | 0.00 | 0.00 | 0.00 | 113.99 | 130.28 |  |  |
| 3 | 1 | 64.82 | 3.25 | 0.00 | 0.00 | 0.61 | 0.00 | 31.66 | 0.00 | 52.77 | 3321.40 | 889.00 |
|  | 2 | 49.03 | 10.24 | 0.00 | 0.00 | 4.27 | 0.00 | 37.06 | 0.00 | 58.67 |  |  |
|  | 3 | 64.86 | 14.65 | 0.00 | 15.35 | 2.51 | 0.00 | 3.12 | 0.00 | 126.34 |  |  |
|  | 4 | 44.65 | 43.84 | 0.00 | 3.89 | 0.61 | 0.00 | 7.42 | 0.00 | 38.36 |  |  |
| 4 | 1 | 75.98 | 0.00 | 0.00 | 0.00 | 0.00 | 23.95 | 0.00 | 0.00 | 69.65 | 1307.10 | 1520.00 |
|  | 2 | 15.20 | 0.51 | 0.00 | 23.52 | 0.00 | 60.49 | 0.00 | 0.00 | 59.06 |  |  |
|  | 3 | 61.74 | 0.00 | 0.00 | 7.19 | 0.00 | 30.63 | 0.00 | 0.00 | 58.10 |  |  |
|  | 4 | 100.00 | 0.00 | 0.00 | 0.00 | 0.00 | 0.00 | 0.00 | 0.00 | 0.00 |  |  |
| 5 | 1 | 0.00 | 1.68 | 0.00 | 0.00 | 2.70 | 0.00 | 95.26 | 0.00 | 12.23 | 1707.30 | 52.00 |
|  | 2 | 53.11 | 1.58 | 6.87 | 0.00 | 0.00 | 0.00 | 38.93 | 46.82 | 63.10 |  |  |
|  | 3 | 72.86 | 6.48 | 18.08 | 0.00 | 0.00 | 0.00 | 0.76 | 172.19 | 170.66 |  |  |
|  | 4 | 27.21 | 6.87 | 7.97 | 0.00 | 1.17 | 0.00 | 57.05 | 72.01 | 112.36 |  |  |
| 6 | 1 | 60.23 | 14.91 | 0.00 | 11.82 | 0.61 | 13.61 | 0.00 | 0.00 | 110.60 | 2048.10 | 921.00 |
|  | 2 | 39.10 | 0.00 | 0.00 | 45.56 | 13.21 | 0.00 | 0.00 | 0.00 | 96.27 |  |  |
|  | 3 | 86.22 | 11.22 | 0.00 | 0.00 | 2.29 | 0.00 | 0.00 | 0.00 | 7.64 |  |  |
|  | 4 | 26.68 | 13.06 | 0.00 | 54.74 | 1.96 | 3.20 | 0.00 | 0.00 | 59.79 |  |  |
| 7 | 1 | 19.07 | 48.95 | 1.74 | 0.00 | 0.00 | 30.54 | 0.00 | 28.60 | 172.63 | 2552.50 | 1848.00 |
|  | 2 | 28.49 | 59.89 | 0.00 | 0.00 | 0.00 | 12.02 | 0.00 | 0.00 | 64.15 |  |  |
|  | 3 | 15.89 | 44.76 | 0.00 | 0.00 | 0.00 | 36.68 | 0.00 | 0.00 | 111.05 |  |  |
|  | 4 | 33.69 | 17.10 | 3.05 | 0.00 | 0.00 | 45.67 | 0.00 | 35.64 | 126.27 |  |  |
| 8 | 1 | 65.36 | 0.00 | 0.71 | 33.44 | 0.00 | 0.00 | 1.22 | 4.07 | 116.54 | 3623.60 | 265.00 |
|  | 2 | 83.60 | 0.00 | 0.00 | 0.00 | 0.00 | 0.00 | 17.40 | 0.00 | 41.84 |  |  |
|  | 3 | 69.88 | 0.00 | 10.08 | 18.89 | 0.00 | 0.00 | 0.00 | 73.83 | 87.07 |  |  |
|  | 4 | 75.45 | 0.00 | 12.99 | 0.31 | 0.00 | 0.00 | 9.07 | 89.15 | 116.66 |  |  |
| 9 | 1 | 47.30 | 18.93 | 0.00 | 22.96 | 0.00 | 11.91 | 0.00 | 0.00 | 101.32 | 1912.30 | 1760.00 |
|  | 2 | 88.23 | 6.37 | 0.00 | 6.21 | 0.00 | 0.00 | 0.00 | 0.00 | 24.42 |  |  |
|  | 3 | 96.63 | 0.00 | 0.00 | 0.00 | 0.00 | 0.00 | 3.16 | 0.00 | 14.80 |  |  |
|  | 4 | 94.60 | 0.05 | 0.00 | 0.00 | 0.00 | 5.20 | 0.00 | 0.00 | 17.32 |  |  |
| 10 | 1 | 17.77 | 5.72 | 1.93 | 30.13 | 0.00 | 43.51 | 0.00 | 58.02 | 151.65 | 2186.20 | 841.00 |
|  | 2 | 29.70 | 10.44 | 0.05 | 0.00 | 0.00 | 57.97 | 0.00 | 1.02 | 141.47 |  |  |
|  | 3 | 48.29 | 5.15 | 0.00 | 0.00 | 0.00 | 46.61 | 0.00 | 0.00 | 45.85 |  |  |
|  | 4 | 17.38 | 22.83 | 0.10 | 2.04 | 0.00 | 61.86 | 0.00 | 3.56 | 247.45 |  |  |

*SD5: Habitat feature covered by receiver towers around the swarming site*

**Table SD5.** Habitat metrics for each antenna of the ten receiver towers installed around a little brown bat swarming site (June-July 2021) in the Outaouais region, Québec, Canada. Towers 9 and 10 were not characterized as they were installed near other hibernaculum and no bats were detected there during the study. Habitat metrics were extracted from 2019 Québec Land Use Classification (10 m resolution; ministère de l’Environnement et de la Lutte contre les changements climatiques, 2022), forest age and harvested forest cover (% harvested) metrics were derived from 2015 Québec Forest Survey data (ministère des Forêts, de la Faune et des Parcs, 2022a, 2022b).

| Tower | Antenna | % Young forest | % Old forest | % Water | % Wetlands | % Urban | % Harvested | % Agriculture | Water edges  (m/ha) | Forest edges  (m/ha) | Distance to roost (m) |
| --- | --- | --- | --- | --- | --- | --- | --- | --- | --- | --- | --- |
| 1 | 1 | 67.71 | 22.40 | 8.93 | 0.00 | 0.20 | 0.00 | 0.00 | 36.81 | 37.82 | 0.00 |
|  | 2 | 60.43 | 38.46 | 0.00 | 0.00 | 1.22 | 0.00 | 0.00 | 0.00 | 6.58 |  |
|  | 3 | 43.86 | 49.72 | 0.00 | 3.25 | 2.68 | 0.00 | 0.00 | 0.00 | 24.23 |  |
|  | 4 | 94.77 | 0.00 | 3.84 | 0.00 | 1.08 | 0.00 | 0.00 | 17.42 | 17.93 |  |
| 2 | 1 | 58.99 | 0.00 | 0.00 | 0.00 | 33.10 | 0.00 | 6.26 | 0.00 | 157.84 | 3434.77 |
|  | 2 | 67.71 | 7.92 | 6.46 | 10.57 | 5.64 | 0.00 | 0.00 | 40.67 | 83.88 |  |
|  | 3 | 20.38 | 52.01 | 3.11 | 3.87 | 2.60 | 0.00 | 18.80 | 23.94 | 73.36 |  |
|  | 4 | 34.38 | 1.32 | 0.00 | 6.00 | 0.76 | 0.00 | 55.87 | 0.00 | 140.32 |  |
| 3 | 1 | 4.60 | 58.73 | 11.90 | 23.96 | 0.00 | 0.00 | 0.00 | 35.61 | 63.58 | 3108.04 |
|  | 2 | 0.00 | 16.56 | 4.48 | 78.20 | 0.00 | 0.00 | 0.00 | 21.40 | 33.62 |  |
|  | 3 | 9.89 | 35.95 | 0.00 | 53.54 | 0.00 | 0.00 | 0.00 | 0.00 | 79.55 |  |
|  | 4 | 50.59 | 47.21 | 0.46 | 1.48 | 0.00 | 0.00 | 0.00 | 4.58 | 10.68 |  |
| 4 | 1 | 23.41 | 61.63 | 11.86 | 0.00 | 4.19 | 0.00 | 0.00 | 24.54 | 38.34 | 4142.39 |
|  | 2 | 43.49 | 51.91 | 0.00 | 2.84 | 1.29 | 0.00 | 0.00 | 0.00 | 51.63 |  |
|  | 3 | 54.90 | 24.35 | 0.00 | 19.88 | 0.00 | 0.00 | 0.00 | 0.00 | 105.98 |  |
|  | 4 | 43.78 | 36.74 | 0.00 | 17.59 | 0.00 | 0.00 | 0.00 | 0.00 | 69.85 |  |
| 5 | 1 | 79.54 | 0.00 | 20.10 | 0.00 | 0.00 | 0.00 | 0.00 | 54.22 | 54.22 | 2712.86 |
|  | 2 | 98.17 | 1.12 | 0.61 | 0.00 | 0.00 | 0.00 | 0.00 | 5.10 | 5.10 |  |
|  | 3 | 52.73 | 20.84 | 26.18 | 0.05 | 0.00 | 0.00 | 0.00 | 42.79 | 43.81 |  |
|  | 4 | 60.67 | 13.11 | 19.26 | 5.84 | 0.00 | 0.00 | 0.00 | 39.96 | 59.94 |  |
| 6 | 1 | 25.66 | 57.08 | 0.00 | 2.65 | 12.50 | 0.00 | 0.00 | 0.00 | 61.73 | 4287.41 |
|  | 2 | 1.12 | 4.59 | 0.00 | 0.00 | 66.92 | 0.00 | 26.88 | 0.00 | 29.47 |  |
|  | 3 | 14.58 | 36.87 | 12.38 | 0.00 | 22.41 | 0.00 | 12.89 | 38.21 | 120.22 |  |
|  | 4 | 27.77 | 66.89 | 3.98 | 0.00 | 0.10 | 0.00 | 0.00 | 27.04 | 25.51 |  |
| 7 | 1 | 59.94 | 34.52 | 0.00 | 0.00 | 4.98 | 0.00 | 0.00 | 0.00 | 34.57 | 4609.75 |
|  | 2 | 91.64 | 6.37 | 0.00 | 0.00 | 1.98 | 0.00 | 0.00 | 0.00 | 49.36 |  |
|  | 3 | 74.17 | 14.32 | 0.00 | 0.00 | 0.00 | 11.42 | 0.00 | 0.00 | 41.82 |  |
|  | 4 | 21.77 | 65.32 | 0.00 | 0.00 | 11.46 | 0.15 | 0.31 | 0.00 | 90.54 |  |
| 8 | 1 | 23.45 | 73.09 | 1.17 | 0.00 | 2.09 | 0.00 | 0.00 | 11.21 | 65.72 | 5592.91 |
|  | 2 | 9.03 | 87.81 | 2.19 | 0.00 | 1.12 | 0.00 | 0.00 | 11.69 | 41.18 |  |
|  | 3 | 0.00 | 48.37 | 48.96 | 0.36 | 1.99 | 0.00 | 0.00 | 48.40 | 101.38 |  |
|  | 4 | 12.47 | 31.55 | 54.46 | 0.00 | 0.00 | 0.00 | 0.00 | 43.30 | 43.30 |  |
